# Medical Matters in Paris

**Published:** 1846-01

**Authors:** F. Campbell Stewart


### MEDICAL EXAMINER,

AND

### RECORD OF MEDICAL SCIENCE.

NEW SERIES—No. XIII.—JANUARY, 1846.

---

### ORIGINAL COMMUNICATIONS.

### *Medical Matters in Paris.*

(Communicated by F. Campbell Stewart, M. D.)

*Paris, October* 2, 1845.

To Dr. Campbell Stewart, New York.

*Chèr et honorable Confrère.*—Some considerable time has elapsed since I wrote to you, and as I am well aware that you take a deep interest in all that is passing with us of a professional nature, I will endeavour to make up for lost time, and henceforth will strive to keep you "*au courant*" with the medical news of the day.

We have recently had several interesting discussions at the Royal Academy of Medicine. Monsieur Collineau offered a report on a memoir of Dr. Giraud, in which the author insists, in the strongest terms, upon the employment of opium, for the treatment of dysentery. In an epidemic of that disease he found all other means to fail, and he considers this narcotic as almost a specific when given in sufficiently large doses. As the article presented no new facts, and no positive proof in the way of detailed cases to support the author's assertions, the Reporter recommended that the memoir should simply be deposited amongst the archives of the Academy.

Monsieur Collineau likewise reported on the merits of an article sent in by Dr. Thomas, on the subject of the treatment of Mucous and Typhoid Fevers; the reading of this report was followed by a spirited discussion amongst different members of the

Academy. As in the memoir, Dr. Thomas recommends most highly the use of blisters. M. Rocheux was of opinion that the conclusions of the report were too favourable; for, said he, "if there is a fact well established in Therapeutics, it is, that blisters, which were so commonly used by the ancients in low fevers, *(fièvres graves)* are both useless and dangerous." Our old friend Louis having been cited by the Reporter as being favourably disposed towards the use of epispastics in these cases, felt himself called upon to state, that M. Collineau had misunderstood the result of his researches on this subject. "I have made," said he, "with all the care and attention of which I am capable, the analysis of a great number of cases of typhoid fever in which I had used blisters. The results of this analysis have led me to think that blisters are useless and even hurtful in such cases—hurtful inasmuch as they are apt to degenerate into ulcers, and thus occasion a tardy convalescence."

You have heard of, and probably know, a Doctor James, editor and proprietor of the "Journal de Vaccine." He is a corresponding member of the Royal Academy, but, greatly to the discredit of the profession and to the annoyance of the Academy, he has, for a number of years, gained his subsistence by selling vaccine matter and practising vaccinations for pay; in a word, he has made vaccination a speciality, and derives a considerable income from the sale of matter, and from contributions intended to promote the circulation of his journal, which is widely distributed about the city and departments. This gentleman was present at a late meeting of the Academy, when a report was read on the subject of vaccination. At the conclusion of the report, Dr. Villeneuve expressed a desire that a recommendation should be made to the minister of the interior, to notify the Prefects and Mayors of the Departments, to be on their guard against "certain assertions" tending to throw discredit upon the matter furnished by the Academy,* as such assertions were intended to benefit individual speculation. Monsieur James immediately took umbrage at this supposed attack upon himself, and demanded permission to speak in reply to it; being only a corresponding member, it was necessary that the President should obtain the consent of the Academy before acceding to his request, and on the question being put, there was an unanimous vote in the negative. A motion was now made to expel M. James, who, very much excited, and regardless of a call to order, was endeavoring to make himself heard. The President, finding that

* Vaccine matter is furnished gratuitously by the Royal Academy of Medicine, to the authorities throughout France, and indeed to any medical man who applies for it. It is always genuine, and may be relied upon with security.

he was unable to controul the rising tumult, left the chair, and put an end to the sitting, when the members separated in great confusion. Thus, you see, our Academy is determined to discountenance charlatanry on all occasions, and will not allow its own members, even, to escape, when they shall be found to merit chastisement.

You will have heard that the great surgeon of Montpellier, Monsieur Lallemand, has determined to make Paris his future residence; he has arrived in the city, and is already established amongst us; rumour says, however, that it is not his intention to practise his profession any longer. He has recently been appointed a member of the Institute, in the department of the Academy of Sciences.

We have but little that is new in surgery.—Monsieur Velpeau repeated a short time since his operation for hydrarthosis of the knee joint, by the injection of a solution of iodine, as he has done for so long a time in hydrocele; the result was again favourable, as the patient has entirely recovered.

Dr. Robert has been using for some time at the Hôpital Beaujon, a preparation of the *nitrate of lead*, for the dressing of foul ulcers; it appears, from his account, to correct the fœtid nature of the discharge which so generally accompanies old and ill-conditioned sores.

The same gentleman likewise speaks favourably of pins for the cure of erectile tumours, when they are allowed to remain in the tumour until they ulcerate their way out; the result appears to have been satisfactory in some cases where the aneurism was small and situated on the face.

There have been one or two changes at the Hospitals. On the death of Breschet, Monsieur Jobert *(de Lamballe)* was appointed to fill his place in the Hôtel-Dieu, with the condition, however, that he should discontinue his public exhibitions of the application of the speculum, which have rendered his clinical lectures so attractive at Saint Louis; considering the condition as unfair and unjust, he declined accepting the nomination. Dr. Philippe Boyer, son of the great surgeon of that name, has been appointed to succeed Breschet, and Monsieur Malgaigne comes in regular turn to take Boyer's place at Saint Louis, where he will doubtless attract many students during the coming session, for besides being one of our most favourite authors, he is an interesting and practical lecturer.

There have been no epidemics of importance this year, and Paris continues to be tolerably healthy.

